# Resonating Behaviour of Nanomachined Holed Microcantilevers

**DOI:** 10.1038/srep17837

**Published:** 2015-12-08

**Authors:** Giancarlo Canavese, Alessandro Ricci, Gian Carlo Gazzadi, Ivan Ferrante, Andrea Mura, Simone Luigi Marasso, Carlo Ricciardi

**Affiliations:** 1 Applied Science and Technology Department, Politecnico di Torino, Torino 10129, Italy; 2 S3 Center, Nanoscience Institute – CNR, Modena 41125, Italy; 3 Mechanical and Aerospace Engineering Department, Politecnico di Torino, Torino 10129, Italy; 4 CNR-IMEM, Parco Area Delle Scienze, 37a, IT, 43124 Parma, Italy

## Abstract

The nanofabrication of a nanomachined holed structure localized on the free end of a microcantilever is here presented, as a new tool to design micro-resonators with enhanced mass sensitivity. The proposed method allows both for the reduction of the sensor oscillating mass and the increment of the resonance frequency, without decreasing the active surface of the device. A theoretical analysis based on the Rayleigh method was developed to predict resonance frequency, effective mass, and effective stiffness of nanomachined holed microresonators. Analytical results were checked by Finite Element simulations, confirming an increase of the theoretical mass sensitivity up to 250%, without altering other figures of merit. The nanomachined holed resonators were vibrationally characterized, and their Q-factor resulted comparable with solid microcantilevers with same planar dimensions.

Functionalized micro and nanocantilevers have been extensively proposed as high performing biological and chemical sensors^1^,^2^,^3^. One of the most investigated and critical figures of merit of a microcantilever (MC) resonator is undoubtedly the mass sensitivity, commonly defined as the frequency change for a unit mass change (Hz/g) or its inverse (g/Hz). For measurements in which the mass-change binding effect is often dominant relative to the stiffness-change one, the theoretical mass sensitivity *S*_n_ can be stated as^4^:


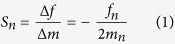


where *f*_*n*_and *m*_*n*_ are the resonance frequency of the n^th^-order mode and the effective mass of the oscillator (for first mode *n* = *1*, *m*_*n*_ = *0.25m*), while *∆m* and *∆f* are the added mass and the correlated frequency shift. For standard microcantilevers used in dynamic mode (length 200–500 μm, width 100 μm, thickness 7 μm), Eq. 1 gives a mass sensitivity of about 1 Hz/pg or lower, for the first mode of vibration.

In bio-chemical sensors field, a larger sensitivity leads to the possibility of detecting smaller masses^5^,^6^,^7^, as well as to the capability of distinguishing smaller variation of target concentration. Both aspects clearly represent strategic guidelines for new biomedical discoveries^8^,^9^,^10^,^11^,^12^,^13^,^14^,^15^.

Different strategies have been adopted to increase mass sensitivity. The two most popular ones are the scale down of cantilever dimensions and the use of higher vibrating modes^16^. The former means the fabrication of nanodevices and consequently it clearly led to the decrease of the resonating mass too^17^,^18^,^19^. Besides, nanodevices are affected by some applicative disadvantages connected to the adsorption-desorption dynamics, as well as to the difficulties in the filtering and the amplification of their read-out signal^20^. Finally, since their dimensions are comparable with the diffusion length of the biomolecules, target binding is not promoted and can result in inhomogeneity of adsorbed molecules on sensor surface^21^,^22^. For what concerns the latter strategy, it has been demonstrated that the use of higher vibration modes enhances the sensitivity in vacuum^23^, air^24^, and liquid^25^, but it is quite challenging to obtain reliable measurements of resonance frequencies and Q factors, especially for those systems that use an out-of-chip readout. As a matter of fact, the oscillation amplitude decreases with the increase of the mode number, leading to a lower signal-to-noise ratio and to an inaccurate estimation of the Q factor^23^,^26^,^27^. This can lead to a decrease of the device resolution, since this last quantity directly depends on Q^4^,^28^. It has been demonstrated, for instance, that the second mode is more sensitive but less precise if compared to the first one^12^. Other solutions to enhance the mass sensitivity are the increase of the cantilever spring constant^29^, by choosing materials with higher Young modulus^30^,^31^,^32^, or the change of the resonator’s shape, through ablation of a part of it^33^,^34^,^35^. While first alternative has intrinsic difficulties in standard micro and nano-fabrication facilities, the presence of necks and slots within the cantilever planar geometry can be detrimental when operating in fluid environment, since a higher device surface exposed to the fluid friction acts negatively on the Q factor^33^,^35^.

In this work we propose an original solution that is able to increase the theoretical mass sensitivity, as well as to overcome the drawback induced by the presence of holes through the cantilever thickness. We designed and fabricated a nanomachined holed MC in which the through holes are made along the cantilever width (Fig. 1(a)): as a consequence, the presence of nanomachined holes perpendicular to the cantilever movement is supposed to minimize the viscous losses induced by the increased surface/fluid friction. This solution effectively acts on both numerator (increasing the frequency) and denominator (lowering the mass) in Eq. 1, while offering other correlated advantages: it allows maintaining a relatively large planar surface to promote target binding, and it is fairly compatible with simple out-of-chip readout systems such as optical or capacitive ones.

It’s worth noting that the here proposed relatively simple nanomachined holed design can be further improved using topological optimization methods, that are well-established and widely adopted in the framework of the mechanics of solids and structures^36^. Each solution should be evaluated in terms of theoretical mass sensitivity, keeping in mind the fabrication constraints. For example, the classical geometry with holes parallel to the beam axis would be unpractical from the nanofabrication point of view. Besides, the aim of the paper is not to discuss all possible optimization strategies, but to show a general approach, based on locally nanomachined microcantilevers, that can be straightforwardly applied to more complicated structures to enhance different mechanical contributions, such as the mass loading respect to the local stiffening.

For easiness, we focus our discussion on the first vibration mode of an isotropic rectangular cantilever beam, with length *l*, width *w*, thickness *t*, elastic modulus *E* and mass density *ρ*. We propose a structure in which the holes are fabricated within the cantilever thickness, and cross the volume of the device along the *y* axis (Fig. 1(a)). We start considering the simplest case, where the hole shape along the *xz* plane is square, with an edge length equal to *h*. Both the distance between the first hole and the cantilever free end, *∆*, as the pitch, *p*, are here kept constant. The resulting cross section area *A(x)* as well as the moment of inertia *I*_*y*_*(x)* are therefore piecewise functions, whose values are *wt* and *1/12wt3* for the *x* coordinate varying between two consecutive holes, and *w(t-h)* and *1/12w(t-h)*^3^ for the *x* coordinate varying within each hole.

Since the modal deflection *u*_*z*_*(x)* derives from a transcendental equation, an analytical solution cannot be obtained. Besides, from the mode shape solutions for vibrating prismatic cantilever beams^37^, an approximated description can be the following^38^:


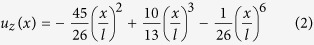


It’s worth noting that the classical beam theory is not any more valid for our current design having abrupt longitudinal variations. Besides, we can use such simple theory as a first screening result, to be finally checked by numerical simulations. Therefore, we assume that the impact of the resulting hole pattern on the mode shape is negligible, so that eq. (2) still holds for our nanomachined holed structures.

To evaluate the fundamental frequency of the system, we employ the Rayleigh-Ritz method^37^, since the equation of motion can’t be analytically solved in a general situation. By employing Euler-Bernoulli beam theory in absence of damping, the equality of the kinetic and potential energy of the structure leads to the following quotient^38^:


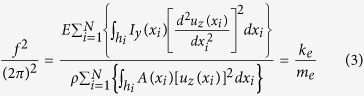


The variables *f*, *k*_*e*_ and *m*_*e*_, are the nanomachined holed cantilever resonance frequency, effective stiffness and effective mass; the suffix *i* indicates each hole, so that *u*_*z*_*(x*_*i*_) is the normalized modal deflection in the center of the i-th hole. *N* is the maximum number of holes that can be realized through the whole length; as construction, 
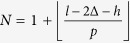
, where 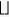 is the floor operator.

We implemented Eqs 2 and 3 in Matlab® as a function of the normalized number of holes *n/N* (so that *n/N* = *1* refers to the maximum of admitted holes, *n/N* = *0* represents the solid cantilever), and calculated the mass sensitivity *S* adding a proof mass of 1fg. To compare them correctly, all the variables *S, f*, *k*_*e*_ and *m*_*e*_ in Fig. 1(b) are normalized to 1 at their maximum value. As easily deducible, both *k*_*e*_ and *m*_*e*_ decrease with an increase of the number of holes *n* (counted starting from the free end): while a mass reduction is a clearly desired effect, a corresponding reduction of the stiffness would be detrimental for the mass sensitivity. However, it is clear in Fig. 1(b) that the slope of the two curves is different, so that the competition between the two effects leads to the existence of an optimized number of holes, for which the resonance frequency *f* and the mass sensitivity *S* are maximized.

Figure 1(c,d) show the dependence of the mass sensitivity *S* to the ratio *t/l*, for two different values of the ratio *h/t*, to prove that a maximum value respect to *n/N* exists for any cantilever geometry. The absolute value of the maximum sensitivity (here normalized as *1* for *n/N* = *0*) markedly depends on the ratio *h/t*, since more and more mass is subtracted if higher is the hole dimension respect to the cantilever geometry.

By considering the trends in Fig. 1b, it is possible to root out alternative way to further increase the theoretical mass sensitivity. Since the acceleration of the structure is proportional to the mode shape, the influence of a mass removal on frequency is highest in proximity of the free end and progressively reduces when approaching the fixed end. A more effective solution to enhance the theoretical mass sensitivity is therefore the fabrication of an hole pattern where the hole width is not constant, but proportional to *u(x)*. In this way the amount of removed mass is raised in proximity of the free end and minimized close to the fixed end, i.e. where the undesired drop of stiffness due to the hole presence is more important. We tested this idea in Matlab® by means of another script which implements Eqs 2 and 3, allowing a variable hole dimension along *x*. The script calculates the number of square holes that maximizes the mass sensitivity and then modifies the hole pattern to obtain further improvement of this parameter. The new hole pattern is obtained from the old one (i.e. regular), by multiplying the hole dimension along *x* for a normalized mode displacement which is equal to 1 in correspondence of the geometric center of the regular pattern. An increase in theoretical mass sensitivity up to 1.25 times the maximum value reached with the regular pattern was achieved.

To experimentally investigate the mechanical behavior of nanomachined holed resonators, the two proposed structures were fabricated and characterized. The fabrication process is based on a Silicon On Insulator (SOI) substrate; it is composed by a bulk wet etching in KOH solution for micro machining the handle layer, and by a reactive ion etching for the device layer. By means of standard UV lithography and BOE solution, the cantilevers were released from the oxide buried layer. More details can be found elsewhere^39^. Then, by using Focus Ion Beam (FIB) technique, resized cantilevers were cut down from the original fabricated ones. The nanomachined holed structure was completed by FIB nanomachining (FEI Strata DB235M Dual Beam system), opened on both the opposite MC sides to guarantee a constant section of the holes, and thus to respect the axial symmetry of the beam. The parameters used for the fabrication of the nanomachined holed cantilever were 300pA of current with 25nm of beam spot size.

We tested two different chips (C1, C2), each one containing two freestanding microbeams with almost identical dimensions: a 3D nanomachined holed MC, and a solid MC as reference. The C1 chip contains the nanomachined holed cantilever with regular hole pattern (Fig. 2(a)), while the C2 chip the nanomachined holed cantilever with the mode shape-dependent hole pattern (Fig. 2(c)). Table 1 reports the geometric parameters of the MCs, as experimentally measured by SEM images. The distance between the first hole and the cantilever free end, *∆*, was fixed to 350 nm. Since all resonators are made of Si (111), their mechanical properties are *E*  =  170GPa, *ν*  =  0.262 and *ρ*  =  2330 Kg/m^3^.

An optical lever set-up was employed to monitor MC resonance curves in vacuum environment (as those reported in Fig. 2(b,d)): briefly, a laser diode was focused on the MC free-end and reflected to a position sensitive device, while actuation was externally achieved by a piezoelectric disk. Experimental details about read-out system can be found elsewhere^9^,^12^.

As further comparison with both the analytical and experimental data, we set up a Finite Element (FE) model of all the aforementioned resonators. We performed eigenfrequency simulations to calculate the first mode resonant frequency of all the cantilevers, as previously described for similar cases^40^,^41^. To conform at best to the real experimental devices, we included in the model also a portion of the silicon substrate on which the cantilevers are clamped. We therefore evaluated the influence of that portion of material, by performing additional simulation runs involving only the microbeam. In all the calculations the mesh was refined until the 99% of convergence was reached.

The experimental data as well as all the theoretical results for the C1 and C2 chips are reported in Table 2 and Table 3, respectively. Each table reports for both reference and nanomachined holed resonators of each chip the resonance frequency as measured experimentally, as calculated by both FE models (with and without the substrate), and as analytically estimated by Eq. 2. Since actual resonators dimensions are uncertainly estimated from SEM images, the direct use of Eq. 1 for comparing the different structures in terms of the mass sensitivity *S* is impractical. Therefore, *S* is calculated by the Matlab® script, adding a proof mass of 1fg. Variation reported in Table 2 and 3 is calculated as relative percentage deviation 
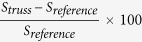
. The same holds for resonance frequencies. A very good agreement between theoretical and experimental results is shown. It is worth noting that the analytical theory slightly overestimates the resonance frequency (i.e. the stiffness). This fact is due to two causes: the ideal boundary condition at the clamped end, and the neglecting of the shear deformation. The former indeed considers the clamp as perfect, i.e. no displacement is allowed in correspondence of it. As a consequence, the overall MC displacement is retrained, which means that the beam behaves as it was stiffer. The latter is an assumption of classical beam theory. While the shear contribution is negligible for a standard beam, the presence of holes within the cross section enhances the shear contribution, which is more and more pronounced with the increase of the ratio *h/t*. Since the actual cantilever out-of-plane displacement is due to both the shear and the bending contributions, neglecting the shear leads to a slight underestimation of the deflection, i.e. to an overestimation of the device stiffness. On the other hand, the FE model including the presence of the substrate exhibits an excellent agreement with experimental data. Both the nanomachined holed resonators show that, with respect to a standard MC, the theoretical mass sensitivity is more than doubled, while the active area is not reduced. In addition, the whole set of data confirm the trend already predicted by the analytical model: if the hole dimension is varied according to the mode shape, the theoretical mass sensitivity is gained respect to the case of a regular pattern of holes of constant dimensions. These facts confirm that the localization of a nanomachined holed structure can be used to effectively tune the theoretical mass sensitivity of a mechanical resonator, without affecting the active area and the resonating characteristics of the device. Figure 2(b,d) reports the experimental curves in vacuum for both reference and nanomachined holed cantilevers of chip C1 and C2, respectively: it is evident that the Q-factors are almost unaffected by the presence of the holes, thus indicating that the local nanomachining is not substantially altering the internal friction during vibration.

Moreover, this strategy can be generally applied to other case studies not analyzed here: for example, the nanomachined holed structure can be differently localized for enhancing mass sensitivity in higher resonant modes, or can be applied to static deflection.

We demonstrated that the localization of a nanomachined holed structure within the lateral cross section of a MC resonator can be used to effectively tune its theoretical mass sensitivity. This idea was firstly investigated by means of a customized analytical model, and then experimentally tested with two different hole patterns. A reduction of the 43% of the cantilever mass with a hole pattern proportional to the first mode shape leads to an increase in resonance frequency of about 49%, that corresponds to an enhancement of theoretical mass sensitivity of nearly 250%. The experimental results have been further confirmed by FE calculations, showing an excellent agreement with them.

Our findings pave the way to a new class of micromechanical devices that can be locally nanomachined to enhance different mechanical contributions, such as the mass loading respect to the local stiffening.

## Additional Information

**How to cite this article**: Canavese, G. *et al.* Resonating Behaviour of Nanomachined Holed Microcantilevers. *Sci. Rep.* **5**, 17837; doi: 10.1038/srep17837 (2015).

## Figures and Tables

**Figure 1:**
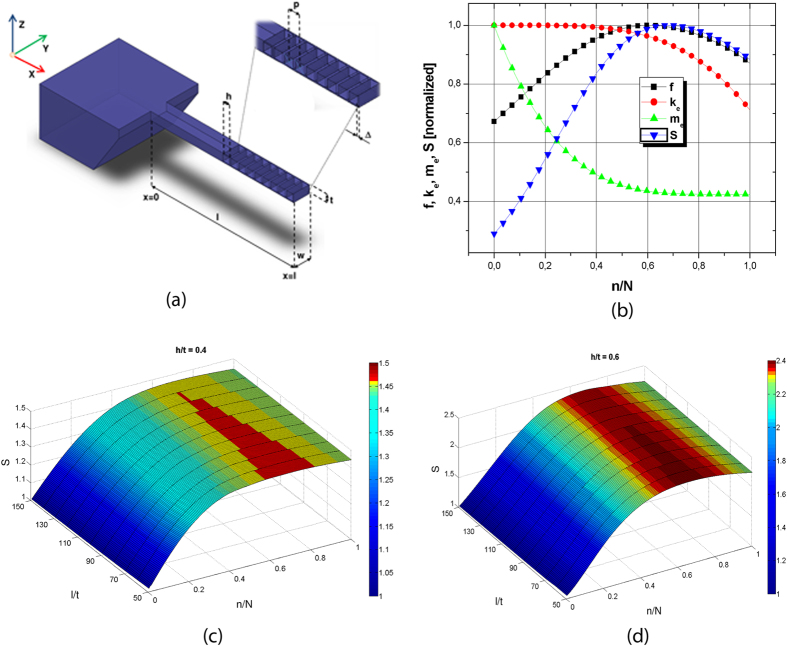
Mechanical characteristics of a nanomachined holed resonator. (**a**) 3D sketch of nanomachined holed microcantilever resonator. (**b**) Normalized resonance frequency f, effective stiffness ke, effective mass me and mass sensitivity S, as a function of normalized number of holes n/N. (**c**,**d**) Normalized mass sensitivity S as a function of l/t and normalized number of holes n/N, for different aspect ratio h/t  =  0.4 and h/t  =  0.6.

**Figure 2:**
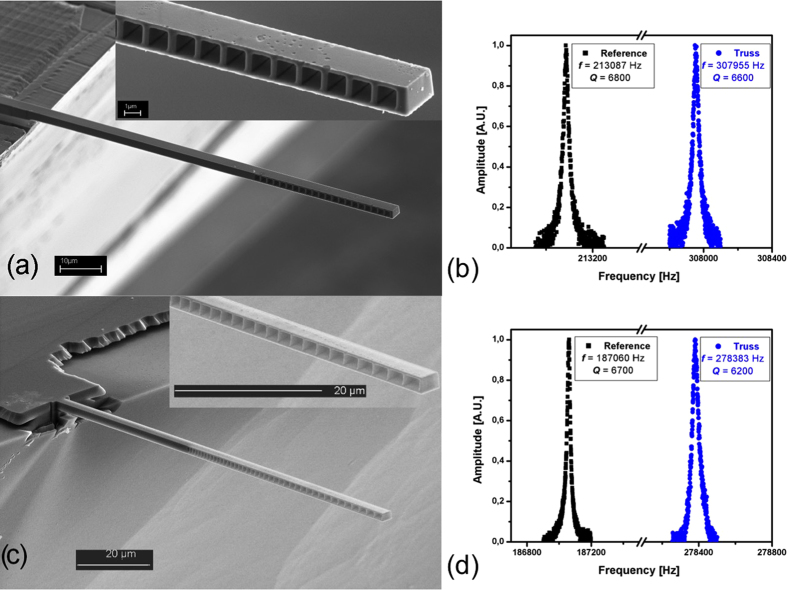
Nanomachined holed resonators: experiments. (**a**) FESEM image of nanomachined holed microcantilever with regular pattern of square holes. (**b**) Plot of the experimental resonance curve in vacuum of reference and nanomachined holed cantilevers of chip C1. (**c**) FESEM image of a nanomachined holed microcantilever with a hole pattern proportional to the first mode shape. (**b**) Plot of the experimental resonance curve in vacuum of reference and nanomachined holed cantilevers of chip C2.

**Table 1:** Geometric parameters of fabricated MCs.

	*l* (μm)	*w* (μm)	*t* (μm)	*n*	*h/t*	*p*(μm)
C1	126.3	3.8	2.5	25	0.68	2.1
C2	126,0	3.9	2.2	41	0.68	1÷2.6

**Table 2:** Experimental and theoretical data of MC with the regular hole pattern.

C1 chip	1^th^ mode frequency (kHz)				S (Hz/pg)
	Exp	FE^a^	FE^b^	Analytical	
Reference	213,1	212,6	216,3	216,3	160
Nanomachined holed	307,9	308,9	314,7	316,5	490
Variation %	44,5%	45,2%	45,5%	46,3%	206,3%

^a^beam + substrate.

^b^beam only.

**Table 3:** Experimental and theoretical data of MC with the hole pattern proportional to the first mode shape.

C2 chip	1^th^ mode frequency (kHz)				S (Hz/pg)
	Exp	FE^a^	FE^b^	Analytical	
Reference	187,1	187,5	191,2	191,2	150
Nanomachined holed	278,4	277,8	283,0	286,2	520
Variation %	48,8%	48,2%	48,0%	49,7%	246.7%

^a^beam + substrate.

^b^beam only.
